# A single freeze-thawing cycle for highly efficient solubilization of inclusion body proteins and its refolding into bioactive form

**DOI:** 10.1186/s12934-015-0208-6

**Published:** 2015-02-22

**Authors:** Xingmei Qi, Yifan Sun, Sidong Xiong

**Affiliations:** Jiangsu Key Laboratory of Infection and Immunity, Institutes of Biology and Medical Sciences, Soochow University, Suzhou, Jiangsu 215123 China

**Keywords:** Bacterial, Recombinant protein expression, Inclusion body, Mild solubilization, EGFP, MMP-12_CAT

## Abstract

**Background:**

Mild solubilization of inclusion bodies has attracted attention in recent days, with an objective to preserve the existing native-like secondary structure of proteins, reduce protein aggregation during refolding and recovering high amount of bioactive proteins from inclusion bodies.

**Results:**

Here we presented an efficient method for mild solubilization of inclusion bodies by using a freeze-thawing process in the presence of low concentration of urea. We used two different proteins to demonstrate the advantage of this method over the traditional urea-denatured method: enhanced green fluorescent protein (EGFP) and the catalytic domain of human macrophage metalloelastase (MMP-12_CAT). Firstly, PBS buffer at pH 8 containing different molar concentration of urea (0-8 M) were used to solubilize EGFP and MMP-12-CAT inclusion bodies and the solubility achieved in 2 M urea in PBS buffer by freeze-thawing method was comparable to that of PBS buffer containing 8 M urea by traditional urea-denatured method. Secondly, different solvents were used to solubilize EGFP and MMP-12_CAT from inclusion bodies and the results indicated that a wide range of buffers containing 2 M urea could efficiently solubilize EGFP and MMP-12_CAT inclusion bodies by freeze-thawing method. Thirdly, the effect of pH and freezing temperature on the solubility of EGFP and MMP-12_CAT inclusion bodies were studied, revealing that solubilization of inclusion bodies by freeze-thawing method is pH dependent and the optimal freezing temperature indicated here is −20°C. Forth, the solubilized EGFP and MMP-12_CAT from inclusion bodies were refolded by rapid dilution and dialysis, respectively. The results showed that the refolded efficiency is much higher (more than twice) from freeze-thawing method than the traditional urea-denatured method. The freeze-thawing method containing 2 M urea also effectively solubilized a number of proteins as inclusion bodies in *E.coli.*

**Conclusions:**

Mild solubilization of inclusion body proteins using the freeze-thawing method is simple, highly efficient and generally applicable. The method can be utilized to prepare large quantities of bioactive soluble proteins from inclusion bodies for basic research and industrial purpose.

## Background

*E.coli* have been most widely used for the production of recombinant proteins in large amounts, due to noticeable advantages such as easy of manipulation, growth on inexpensive carbon sources and fast in generation of a recombinant protein [1,2]. However, heterologous protein over-expression in *E.coli* often leads to the target protein accumulating in dense water-insoluble aggregates, known as inclusion bodies, only about 30% of them were expressed in soluble forms [3]. In general, to obtain soluble active proteins from inclusion bodies, the inclusion bodies are solubilized by the use of high concentration of denaturing reagents such as urea or guanidinium chloride, and then followed by a step of refolding process using various refolding techniques involving slow removal of the denaturant agents [4-7]. The use of the strong denaturants results in the loss of secondary structure leading to the random coil and exposure of the hydrophobic surface, which is considered to be the main reason for the poor recovery of bioactive protein from the inclusion bodies [7-9]. In most cases, the overall yield of bioactive protein from inclusion bodies is around 15–25% of the total protein and amount of precipitation is formed during the refolding process [8]. Also, renaturation of most proteins is performed at rather low protein concentrations (10–100 μg/mL), which is not very convenient on the industrial scale [10]. Despite extensive experimental and theoretical work has been made to produce soluble proteins from inclusion bodies, refolding of the denatured proteins remains a major bottleneck in supplying recombinant proteins for research and industrial applications [5,11-14]. It has been widely documented that inclusion bodies are pure, structurally organized, mechanically stable and biocompatible protein deposits and have native-like secondary structure [15-19]. Mild solubilization of inclusion body aggregates has attracted attention in recent days, it allows for preservation of existing native-like secondary structure of proteins, reduces protein aggregation during refolding and allows for recovery of high amount of bioactive proteins from inclusion bodies [20,21]. In recent years, many novel solubilization methods without using high concentration of denaturing reagents have been developed for solubilization and recovery of bioactive protein form inclusion bodies [13,14,22-24].

**Figure 1:**
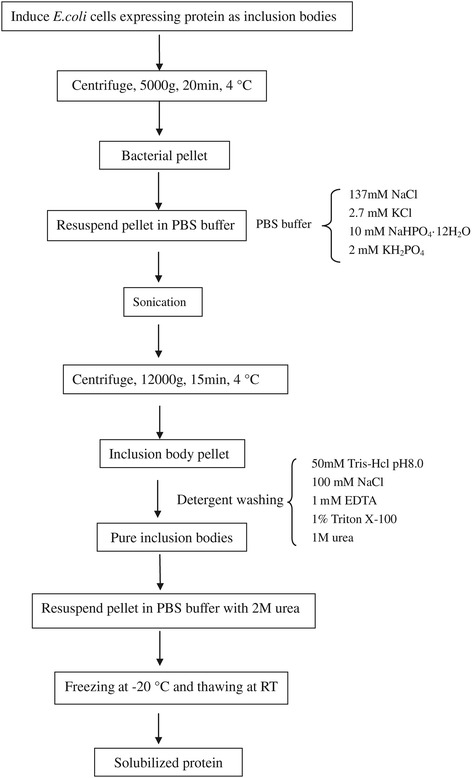
**Scheme for the efficient production of soluble protein from bacterial inclusion bodies using freeze-thawing method.**

Proteins are routinely stored as frozen solutions with the aim of long-term stability. It has been reported that freezing and thawing of protein solutions can cause protein denaturation and destabilize the tertiary structure of proteins with retention of secondary structure [25-27]. There are no reports on solubilization and subsequent refolding of inclusion body proteins using freeze-thawing process. In this study, we explored the possibilities of solubilizing inclusion bodies expressed in *E.coli* with freeze-thawing process in presence of low concentration of urea, which was referred to as freeze-thawing method. In this procedure, the inclusion bodies are suspended in PBS buffer containing 2 M urea, followed by freezing at −20°C and thawing at room temperature, and centrifuged to collect the supernatant. Such an inclusion body protein solubilization strategy is depicted in Figure 1 and has been successfully applied for solubilizing several recombinant proteins expressed as inclusion bodies in *E.coli*, thus providing a novel method for solubilization of a large range of inclusion body proteins in the presence of low concentration of denaturants. Such mild denaturing conditions facilitate the subsequent refolding and purification procedure resulting in high recovery of bioactive protein.

## Results

### Reason for choice of two representative proteins

To test solubilization capacity and refolding efficiency by the freeze-thawing method and to compare with the traditional urea-denatured method, we analyzed two proteins, EGFP and MMP-12_CAT. EGFP (27 kDa) could be produced in both active form and inactive inclusion bodies in *E. coli* expression system depending on inducing protein over-expression conditions [28,29]. Meanwhile, the green fluorescence of EGFP can be easily measured by fluorescence spectrometry and can be used as a sensitive assay to monitor protein folding [28]. Human macrophage metalloelastase (MMP-12) belongs to the family of zinc endoproteases that have the ability to degrade all of the major protein constituents of the extracellular matrix. MMP-12 is secreted from cells as a 54-kDa proenzyme or zymogen [30]. Upon activation, MMP-12 cleaves both its N-terminal prodomain and its C-terminal hemopexin-like domain, resulting in a mature, active, 22 kDa catalytic domain [30,31]. Previous study have shown that the catalytic domain of human macrophage metalloelastase (MMP-12_CAT) are expressed at high levels in *E. coli* only as inclusion bodies, and its active form could be obtained by using high concentration of denaturing reagents denaturing and refolding [3,31]. Also, the activity of MMP-12_CAT was able to determine by using the fluorometric substrate Mca-PLGLEEA-Dpa-NH2 which is commercial available [32]. So, we can compare the solubilization capacity and refolding efficiency of MMP-12_CAT from freeze-thawing method with that from traditional urea-denatured method.

### Isolation of EGFP and MMP-12_CAT inclusion bodies

**Figure 2:**
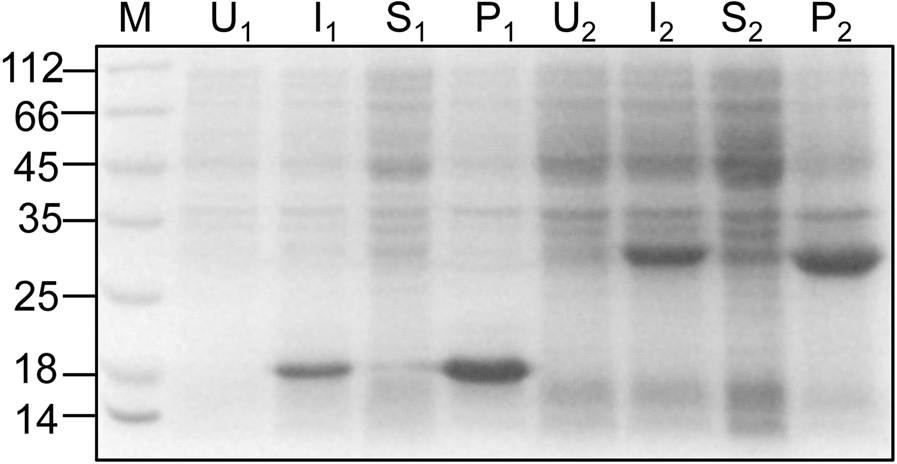
**SDS-PAGE analysis of EGFP and MMP-12_CAT over-expression in** ***E.coli*** **as inclusion bodies.** *E. coli* BL21 (DE3) cells harbouring expression vector of EGFP and MMP-12 catalytic domain, respectively, were induced with IPTG at 37°C for 4 hours. Equivalent numbers of uninduced (U_1_ and U_2_) and induced cells (I_1_ and I_2_) are shown. Equivalent proportions of the soluble fractions (S_1_ and S_2_) and insoluble inclusion bodies (P_1_ and P_2_) are shown. The number 1 represents *E.coli* cells harbouring MMP-12_CAT expression vector and number 2 represents *E.coli* cells harbouring EGFP expression vector. MMP-12_CAT runs as the band of 22 kDa and EGFP as the band of 27 kDa in accordance with their expected size. Molecular weight markers (M) are shown and their sizes in kilodaltons are indicated on the left.

**Figure 3:**
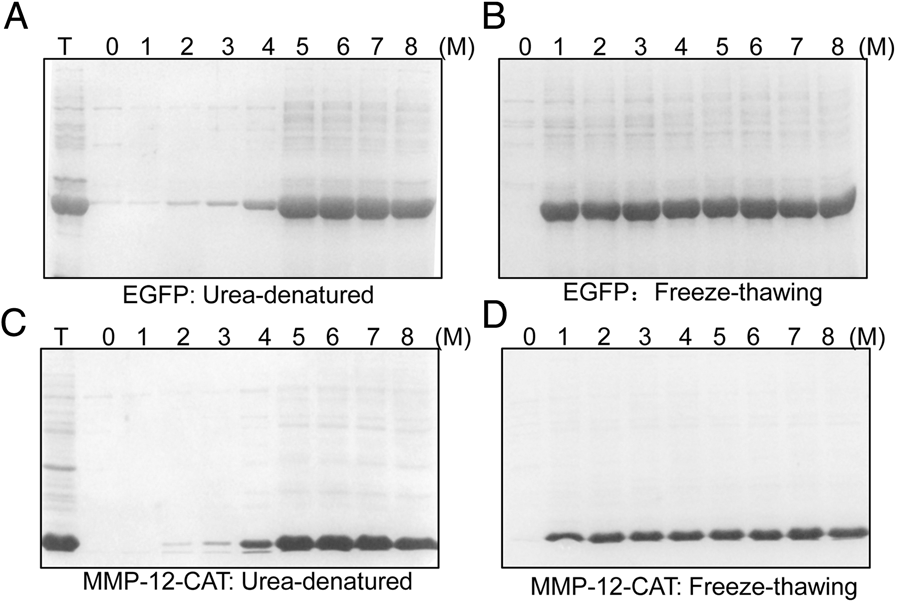
**SDS-PAGE analysis of EGFP and MMP-12_CAT solubilization from inclusion bodies in PBS buffer containing different molar concentration of urea by two different methods.** The same amount of EGFP and MMP-12_CAT inclusion bodies was suspended in PBS buffer at pH 8 containing different molar concentration of urea (0-8 M) and solubilized by freeze-thawing method and traditional urea-denatured method. SDS-PAGE analysis of the solubilization of EGFP from inclusion bodies in different molar concentration of urea by traditional urea-denatured method **(A)** and freeze-thawing method **(B)**; SDS-PAGE analysis of the solubilization of MMP-12_CAT from inclusion bodies in different molar concentration of urea by traditional urea-denatured method **(C)** and freeze-thawing method **(D)**. T stands for cleaned total inclusion bodies.

The EGFP and MMP-12_CAT proteins were efficiently expressed using their expression vector in *E.coli*. As previously reported, EGFP could be produced in both soluble form and insoluble inclusion bodies strongly depends on the growth temperature [3,28], with growth at a low temperature of 16°C resulting into the highest percentage of correctly folded protein (data not shown); at high growth temperature (37°C), EGFP was found to be mainly as insoluble inclusion bodies (Figure 2). In the case of MMP-12_CAT, all of the protein was only found in the insoluble pellet after cell-lysis and not in the soluble fraction (Figure 2). After centrifugation of the cell-lysate and removal of the supernantant, the pelleted crude inclusion bodies were mainly contaminated by the cell envelope and the membrane cellular debris. Extensive washing with detergents containing buffer such as Triton X-100 and/or low concentrations of chaotropic compounds helped in removing the majority of the contaminants and resulted in pure inclusion bodies. At this stage, inclusion bodies purity is more than 90% and therefore less downstream steps are necessary to purify the protein (Lane T, Figure 3). The purified inclusion bodies containing mostly EGFP and MMP-12_CAT were used for subsequent solubilization and refolding.

### Solubilization of EGFP and MMP-12_CAT from inclusion bodies

#### Comparative solubilization of inclusion bodies by two different methods

The solubilization power of freeze-thawing method was compared with that of urea-denatured method. Purified inclusion bodies of EGFP and MMP-12_CAT (10 mg/ml wet weight for final concentration) were solubilized in PBS buffer at pH 8 containing different molar concentration of urea. SDS–PAGE analysis of the solubilized supernatants of these inclusion bodies and protein concentration was measured by Micro BCA Protein Assay Kit (Thermo), BSA as standard protein (Figure 3 and Table 1). It was observed that EGFP and MMP-12_CAT inclusion bodies could be solubilized in PBS buffer containing 5–8 M urea by traditional urea-denatured method, while PBS buffer containing lower concentration of urea (0–3 M) did not solubilize EGFP and MMP-12_CAT inclusion bodies (Figure 3A and C). There was slight solubilization of EGFP and MMP-12_CAT from inclusion bodies in presence of 4 M urea (Lane 4, Figure 3A and C). For freeze-thawing method, PBS buffer containing 1–8 M urea could highly solubilize EGFP and MMP-12_CAT inclusion bodies (Figure 3B and D). Maximum solubilization of EGFP and MMP-12_CAT from inclusion bodies was achieved in PBS buffer containing 2 M urea by freeze-thawing method and higher concentration of urea almost did not further solubilize GFP and MMP-12_CAT from inclusion bodies. It was also observed that PBS buffer alone did not solubilize inclusion body proteins (Lane 0, Figure 3B and D). The results indicated that the solubility achieved in 2 M urea in PBS buffer by freeze-thawing method was comparable to that of PBS buffer containing 8 M urea by traditional urea-denatured method.

**Table 1:** **Comparative the solubilized protein of EGFP and MMP-12_CAT from inclusion bodies in different concentration of urea by two different methods**

**Composition of buffer**	**Protein Conc. (ug/ml)**			
	**EGFP**		**MMP-12_CAT**	
	**Urea-denatured**	**Freeze-thawing**	**Urea-denatured**	**Freeze-thawing**
PBS, pH 8, 0 M urea	115	241	58	202
PBS, pH 8, 1 M urea	116	987	101	1399
PBS, pH 8, 2 M urea	164	1167	109	1594
PBS, pH 8, 3 M urea	252	1274	145	1623
PBS, pH 8, 4 M urea	432	1314	505	1601
PBS, pH 8, 5 M urea	1335	1289	1837	1580
PBS, pH 8, 6 M urea	1371	1331	1819	1292
PBS, pH 8, 7 M urea	1262	1187	1755	1861
PBS, pH 8, 8 M urea	1246	1181	1681	1523

#### Solubilization of inclusion bodies in different solvents

**Figure 4:**
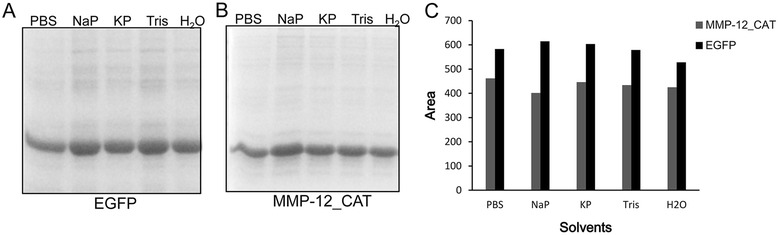
**Effect of different solvents on the solubility of EGFP and MMP-12_CAT inclusion bodies.** A fixed amount of EGFP and MMP-12_CAT inclusion bodies was suspended in different solvents containing 2 M urea and solubilized by freeze-thawing method. **(A)** SDS-PAGE analysis of effect of different solvents on the solubility of EGFP inclusion bodies. **(B)** SDS-PAGE analysis of effect of different solvents on the solubility of MMP-12_CAT inclusion bodies. PBS, PBS at pH 8 with 2 M urea; NaP, 20 mM sodium phosphate at pH 8 with 2 M urea; KP, 20 mM potassium phosphate at pH 8 with 2 M urea; Tris, 20 mM Tris at pH 8 with 2 M urea; H_2_O, deionized water at pH 8.5 with 2 M urea. **(C)** Protein bands were quantitated by densitometric analysis using ImageJ Software.

The solubilizing effect of PBS buffer at pH 8 containing 2 M urea on EGFP and MMP-12_CAT inclusion bodies was compared with different solubilizing buffers as described under Materials and Methods. It was observed that the solubility achieved in 20 mM NaP, 20 mM KP, 20 mM Tris–HCl, and deionized water containing 2 M urea was comparable to that of PBS buffer containing 2 M urea by freeze-thawing method (Figure 4). The results indicated that a wide range of buffers containing 2 M urea by freeze-thawing method could efficiently solubilize EGFP and MMP-12_CAT proteins from inclusion bodies and the solubilize efficiency was comparable to that of traditional urea-denatured method.

#### Solubilization of inclusion bodies at different pH

**Figure 5:**
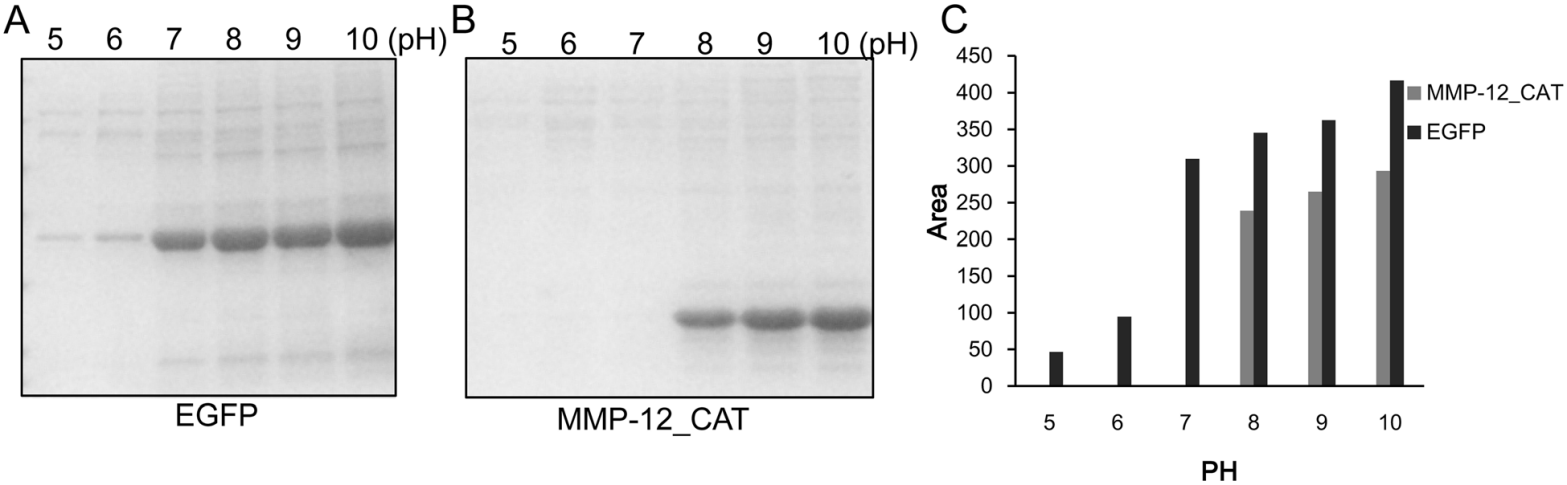
**Effect of pH on the solubility of EGFP and MMP-12_CAT inclusion bodies by freeze-thawing method.** A constant amount of EGFP and MMP-12_CAT inclusion bodies was solubilized at different pH in 20 mM potassium phosphate buffer containing 2 M urea by freeze-thawing method. **(A)** SDS-PAGE analysis of effect of pH on the solubility of EGFP inclusion bodies. **(B)** SDS-PAGE analysis of effect of pH on the solubility of MMP-12_CAT inclusion bodies. **(C)** Protein bands were quantitated by densitometric analysis using ImageJ Software.

The low concentration of potassium phosphate buffer with minmizing pH change during the freezing process was chosen for study the effect of pH on the solubility of inclusion bodies [25,27]. The purified EGFP and MMP-12_CAT inclusion bodies were solubilized at different pHs (pH 5–10) in 20 mM potassium phosphate buffer containing 2 M urea and percentage solubilization was monitored (Figure 5). Solubilization of EGFP and MMP-12_CAT from inclusion bodies was observed by increasing the pH from 5 to 10. SDS–PAGE showed that EGFP inclusion bodies could be solubilized in potassium phosphate buffer at pH 7–10 containing 2 M urea and very low amount of EGFP inclusion bodies could be solubilized at low pH (pH 5 and 6). In the case of MMP-12_CAT, potassium phosphate buffer at pH 5–7 containing 2 M urea did not solubilize protein from inclusion bodies. In contrast, potassium phosphate at high pH (pH 8–10) containing 2 M urea solubilized maximum amount of protein form the inclusion bodies of MMP-12_CAT. The results indicated that inclusion body solubilization by freeze-thawing method in the presence of low concentration of urea is pH dependent and optimum pH conditions must be determined for each protein.

#### Effect of freezing temperature

**Figure 6:**
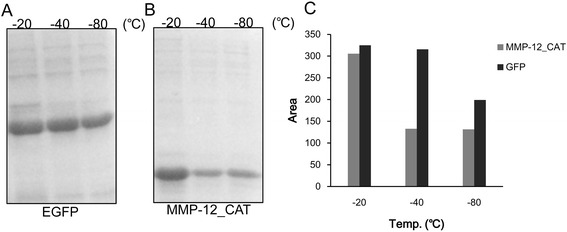
**Effect of freezing temperature on the solubility of EGFP and MMP-12_CAT inclusion bodies by freeze-thawing method.** The same weight of EGFP and MMP-12_CAT inclusion bodies was solubilized in 20 mM potassium phosphate buffer at pH 8 containing 2 M urea and freezing at different temperatures by freeze-thawing method. **(A)** SDS-PAGE analysis of effect of freezing temperature on the solubility of EGFP inclusion bodies. **(B)** SDS-PAGE analysis of effect of freezing temperature on the solubility of MMP-12_CAT inclusion bodies. **(C)** Protein bands were quantitated by densitometric analysis using ImageJ Software.

The solubilizing effect of freezing temperature on EGFP and MMP-12_CAT inclusion bodies was performed in 20 mM potassium phosphate buffer at pH 8 containing 2 M urea. As shown in Figure 6, EGFP inclusion bodies could be solubilized in potassium phosphate buffer at pH 8 containing 2 M urea at freezing temperature −20°C, −40°C and −80°C, which indicated that the freezing temperature has little effect on the solubilization efficiency of EGFP inclusion bodies. In contrast, potassium phosphate buffer containing 2 M urea solubilized very low amount of MMP-12_CAT inclusion bodies while freezing at −40°C and −80°C (Figure 6). The results indicated that the effect of freezing temperature on the solubility of inclusion bodies depends on the protein and the optimal freezing temperature indicated here is −20°C.

### Comparative refolding yield of EGFP and MMP-12_CAT from two different methods

**Figure 7:**
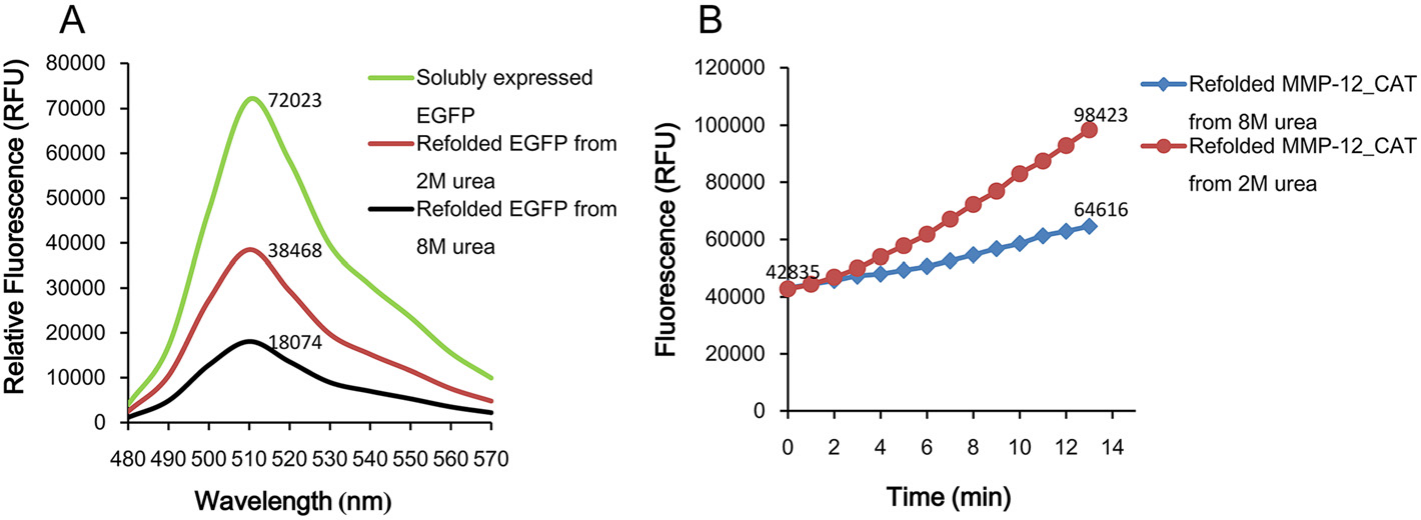
**Activity assay of refolded EGFP and MMP-12_CAT from inclusion bodies. (A)** Fluorescence spectra of refolded EGFP by freeze-thawing method (Refold EGFP from 2 M urea) and traditional urea-denatured method (Refold EGFP from 8 M urea) compared to native EGFP (Solubly expressed EGFP). **(B)** Fluorescence increase for the degradation of the Mca-PLGLEEA-Dpa-NH2 (8 uM) by refolded MMP-12_CAT (5 nM) at 25°C from freeze-thawing method and traditional urea-denatured method.

In the case of EGFP, fluorescence was taken as a sign of a correctly folded and oxidized protein. During the repaid dilution step, there was no precipitation observed. The fluorescence spectrum of EGFP refolded from inclusion bodies was compared to the EGFP soluble expression at the concentration of 0.5 mg/ml. As shown in Figure 7A, the maximal fluorescent intensities at 510 nm were 72023 RFU for the soluble expressed EGFP, 38468RFU for the refolded EGFP from the freeze-thawing method with 2 M urea and 18074RFU for refolded EGFP from the traditional urea-denatured method with 8 M urea. Therefore, when comparing the fluorescence emission at 510 nm of refolded EGFP to that of EGFP from soluble expressed, it showed that the solubilizd EGFP protein from freeze-thawing method had 53.4% recovery ratio comparing to 25.1% from the traditional urea-denatured method.

In contrast to the direct dilution method, solubilized MMP-12_CAT was refolded by the utilization of dialysis as described under Materials and Methods, which allowed the change from denaturing to native buffer conditions occurs gradually. After dilution to the refolding buffer without urea, appreciable precipitation was observed. After centrifugation, the supernatant containing refolded MMP-12_CAT was collected and performed the activity assay. The biochemical activity of the refolded MMP-12_CAT from freeze-thawing method was measured as △RFU/△T at 25°C is 3970.6 RFU, comparable with 1555.8 RFU of the refolded MMP-12_CAT from traditional urea-denatured method (Figure 7B).

In summary, the comparison studies of EGFP and MMP-12_CAT demonstrated that the refolding yield with the freeze-thawing method is significantly better (more than twice) than the traditional urea-denatured method.

### Existence of native-like secondary structure of EGFP by freeze-thawing method

**Figure 8:**
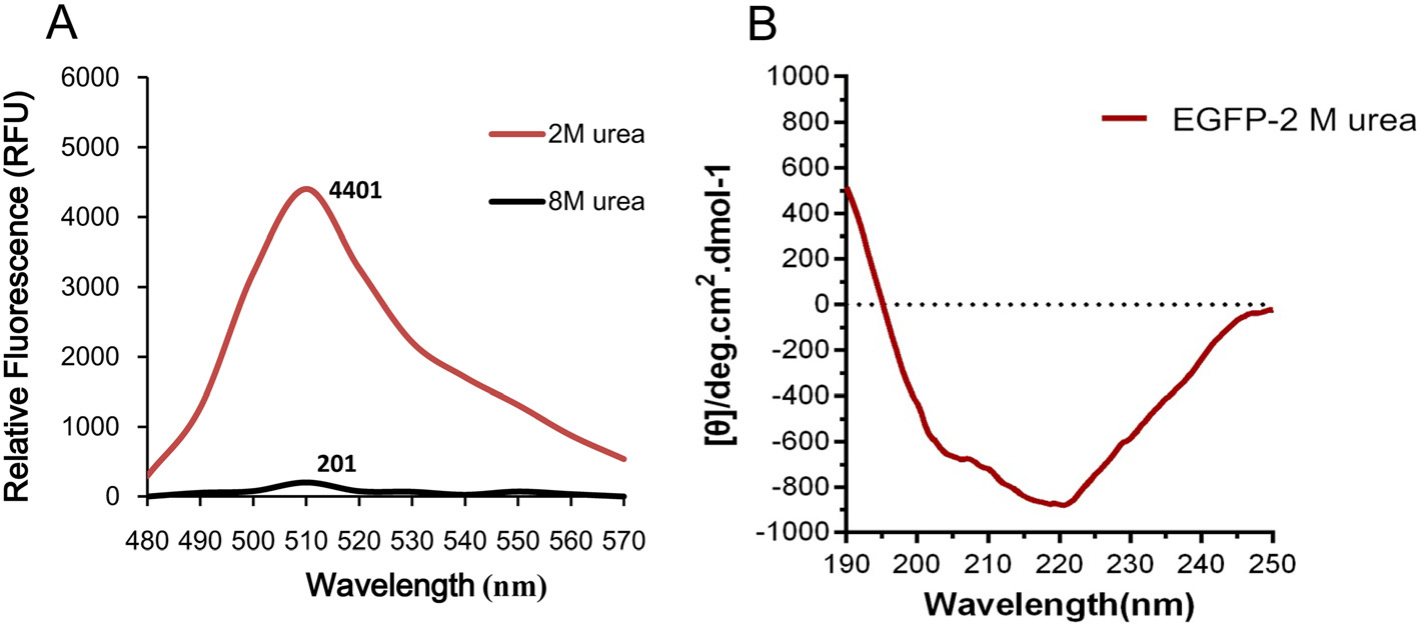
**Fluorescence and CD spectra of solubilized EGFP from inclusion bodies. (A)** Fluorescence spectra of solubilized EGFP from freeze-thawing method (2 M urea) and traditional urea-denatured method (8 M urea). **(B)** Far UV CD spectra of solubilized EGFP by freeze-thawing method.

To validate the mild solubilization strategy of inclusion body protein by freeze-thawing method, the native like secondary structure of solubilized EGFP was monitored by CD and fluorescence spectroscopy (Figure 8). As shown in Figure 8A, fluorescence of two solubilized EGFP samples by two different methods was recorded. The maximal fluorescent intensities at 510 nm were 4401RFU with 2 M urea from the freeze-thawing method (2 M urea), suggesting that EGFP proteins were solubilized as both active and inactive forms by freeze-thawing method. The fluorescence intensity of denatured EGFP from traditional urea-denatured method (8 M urea) was almost overlapped into a horizontal line, which indicated that the solubilized EGFP from inclusion bodies was as inactive form. This was further supported by the CD spectra of solubilized EGFP by freeze-thawing method (Figure 8B). It was observed that EGFP in presence of 2 M urea has native-like secondary structure as observed with the native EGFP.

### General applicability of the freeze-thawing method

**Figure 9:**
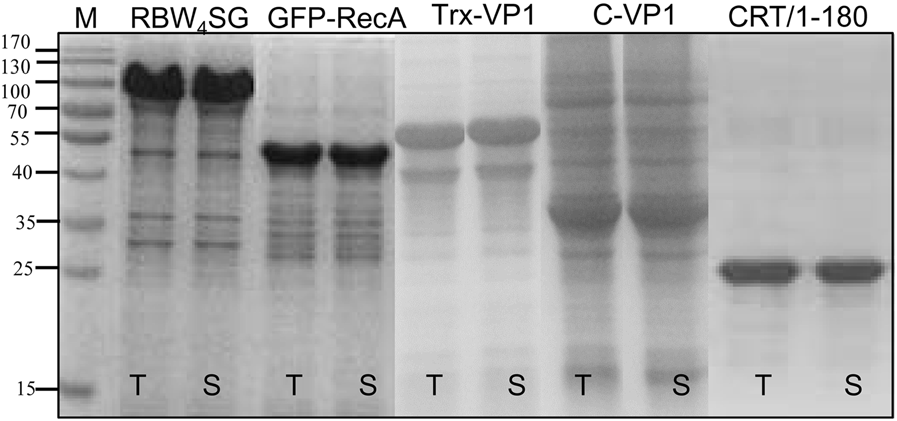
**SDS–PAGE analysis of five solubilized proteins from inclusion bodies.** Five inclusion body proteins were suspended in PBS buffer (pH 8) containing 2 M urea and followed by freezing at −20°C and thawing at room temperature. Lane M is marker (170, 130, 100, 70, 55, 40, 35, 25, 15 kDa), T stands for cleaned total inclusion bodies, S stands for solubilized protein supernatants from inclusion bodies.

To further investigate the universal applicability of the freeze-thawing method, inclusion bodies of five more recombinant proteins (over-expressed in *E.coli* only as inclusion bodies) with different molecular weight and protein physicochemical properties were prepared using the same protocol as EGFP and MMP-12_CAT inclusion bodies. The five inclusion body proteins were named as RBW_4_SG (95 kDa), GFP-RecA (55 kDa), Trx-VP1 (56 kDa), CRT/1-180 (20 kDa) and C-VP1 (34 kDa). C-VP1 is a circular protein produced by covalent cyclization of its backbone by protein trans-splicing, while the others are linear protein forms. Inclusion bodies were suspended in PBS buffer at pH 8 containing 2 M urea and frozen at −20°C overnight, then thawed at room temperature. Centrifuged and collected the supernatants to analyze by SDS-PAGE to check the solubility of these inclusion bodies (Figure 9). It was observed that all the five inclusion bodies could be solubilized using the freeze-thawing method in presence of 2 M urea and the overall yield of soluble proteins was nearly 100%. This indicated that the freeze-thawing method could be used for solubilization of a wide range of recombinant proteins expressed as inclusion bodies in *E.coli*.

## Discussion

Recently study showed that proteins inside inclusion body aggregates have native-like secondary structures [15,16]. It is assumed that retaining of this native-like secondary structure using mild solubilization conditions will help in improved recovery of bioactive protein in comparison to solubilization using a high concentration of chaotropic agent [20,22,24]. In order to develop an efficient and economic mild solubilization conditions to solubilize inclusion bodies and to get higher final refolding yields of bioactive proteins, various mild solubilization agents have been tried. Extreme pH (≤2.6 or ≥ 12) [21,22], has been used to solubilize inclusion bodies. Although the extreme pH solubilization method is attractive for its simplicity and low cost, it may cause irreversible chemical modifications to the protein and not be applicable to most pharmaceutical proteins [5]. Detergents such as sodium dodecyl sulfate (SDS) and n-cetyl trimethylammonium bromide (CTAB) were also been used to solubilize inclusion bodies [28,33,34], however, the use of detergents as solubilizing agents may interfere with downstream chromatographic steps and extensive washing process may be needed to remove solubilizing detergents. In this report, we described a novel way of mild solubilization of inclusion bodies by using low concentration of urea combined with a freeze-thawing process. Inclusion bodies of EGFP and MMP-12_CAT were isolated to more than 90% purity by extensive washing. As the inclusion bodies consisted of almost target protein only, solubilization and refolding were carried out without further purification. The efficiency of solubilization inclusion bodies in several solvents containing 2 M urea by freeze-thawing method (freezing at −20°C and thawing at room temperature) was found to be comparable with that of 8 M urea. This suggested that a wide range of solvents could efficiently solubilize inclusion bodies by freeze-thawing method and appropriate buffer can be chosen to solubilize inclusion bodies according to the downstream applications. Our results also showed that the yield of bioactive EGFP and MMP-12_CAT from inclusion bodies were significantly better than the traditional urea-denatured method. The freeze-thawing method combined 2 M urea also solubilized many inclusion body proteins expressed in *E. coli* only as inclusion bodies indicating that the method has the capacity to solubilize different types of inclusion body aggregates.

Since potassium phosphate buffer with a low concentration does not change its pH significantly (<1 Unit) during freezing, so 20 mM potassium phosphate was chosen to study the effect of pH and freezing temperature on the solubility of EGFP and MMP-12_CAT inclusion bodies [25,27,35]. 20 mM potassium phosphate at alkaline pH (8–10) in the presence of low concentration of urea (1–2 M) was responsible for higher solubilization of EGFP and MMP-12_CAT inclusion bodies. Low concentration of urea and alkaline pH are both essential factors for highly solubilization of inclusion bodies by freeze-thawing method. Increased solubility of EGFP and MMP-12_CAT could be an effect of both urea and pH, indicating the existence both ionic and hydrophobic interactions in the inclusion bodies [22]. Since proteins could not be denatured at such a low concentration of urea and at a mild alkaline pH (8–10), urea was probably only serving the purpose of physical separation of the molecules by disrupting the hydrophobic interactions of protein molecules [36,37]. The alkaline pH distant from the isoelectric point of the protein of buffer has a crucial role in destabilizing the inclusion body aggregation, thus rendering them soluble in the presence of very low concentrations of denaturants.

The mechanism of freezing and thawing on protein stability has been extensively studied [25-27,35,38,39]. Previous study show that freezing can induce several stresses during the freezing process that are capable of denaturing proteins, such as cold temperature, ice crystals formed, the buffer salts concentration, and resultant pH changes. Since 20 mM potassium phosphate buffer has little change in its pH during freezing, hence pH -induced solubilization of inclusion bodies is excluded during freezing process. Meanwhile, deionized water just containing 2 M urea could efficiently solubilize EGFP and MMP-12_CAT inclusion bodies, which also exclude the salt concentration-induced solubilization of inclusion bodies. So, the main force of solubilization of inclusion bodies by freeze-thawing method should mainly indicate towards the stress of cold temperature and ice crystals formed during freezing process. Our study also showed that the freezing temperature had big effect on the solubility of MMP-12_CAT inclusion bodies.

Multimeric proteins have been observed to undergo freezing-induced dissociation but maintain their native secondary structure [25,35,40-42]. Inclusion bodies are composed of aggregates of partially folded protein intermediates and the proteins inside inclusion body aggregates have extensive native-like secondary structure. Inclusion bodies can be seen as large multimeric proteins composed of numbers of monomers, the large multimeric protein are prone to dissociation to subunits during freezing and thawing process, and the dissociated monomers retaining the native-like secondary structure maintain soluble form in the presence of low concentration of urea and at alkaline pH. Once the inclusion body proteins are solubilized under such mild conditions, the subsequent refolding and purification are easier resulting in high recovery of the bioactive protein. Several groups have also reported that properly folded protein might be trapped inside inclusion bodies [29,43-45]. This protein can be released from inclusion bodies with mild detergents under non-denaturing conditions. In our freeze-thawing method, the active EGFP protein was also obtained from such inclusion bodies without any renaturation procedure. However, in current study we did not investigated the effect of different solvents on the secondary structure of solubilized proteins and the yields of recovering bioactive protein form inclusion bodies. Also, the effect of cooling and warming rate on protein secondary structure and recovery efficiency during the freezing and thawing process were needed to be study. Further studies to optimize the conditions for maximizing refolding proteins from inclusion bodies are necessary.

## Conclusion

In summary, the freeze-thawing method is much more convenient, simply, time-saving and efficient compared to the traditional urea-denatured method. Solubilization of inclusion body proteins at such a mild condition preserves the native-like secondary structure of the solubilized proteins and facilitates improved recovery of the bioactive protein. The freeze-thawing method also has the capacity to solubilize a board range of inclusion body proteins. With such multiple advantages, freeze-thawing method might be a viable alternative to high concentration of denaturing agents based solubilization of inclusion body protein. As the yield of recovery of bioactive protein is high using freeze-thawing method, such technique can be applied for maximizing the recovery of proteins from inclusion bodies expressed in *E.coli,* especially for large scale for industrial processes.

## Materials and methods

### Expression of EGFP as soluble form and insoluble inclusion bodies in *E.coli*

The gene encoding enhanced GFP was cloned from pEGFP-N1 plasmid (BD Clontech, Palo Alto, CA) and inserted into pET28a expression vector and transferred into BL21 (DE3). When the cells were grown in LB medium at 37°C with the absorbance at 600 nm (OD 600) reached 0.8, a final concentration of 1 mM IPTG was added to induce the EGFP soluble expression at 16°C for another 16 hours, otherwise insoluble form was induced at 37°C for 4 hours. For EGFP soluble expression, the cells were harvested at 5,000 g at 4°C for 20 minutes and resuspended in 30 ml of PBS buffer (137 mM NaCl, 2.7 mM KCl, 10 mM Na_2_HPO_4_ · 12H_2_O, 2 mM KH_2_PO_4_, pH 8.0), and then lysed by sonication on ice (Branson Sonifier 450, USA). The lysate was centrifuged at 12,000 g at 4°C for 20 minutes. The clarified supernatant was collected and purified by Ni-NTA affinity chromatography (GE healthcare) following the instructions. For insoluble expression, cells were harvested at 5,000 g at 4°C for 20 minutes and the pellets were stored at −20°C.

### Expression of catalytic domain of MMP-12 in *E.coli*

The gene coding for the catalytic domain of human MMP-12 (Gly106 to Asn268) was cloned into *BamHI* and *NdeI* sites of a T7-based expression plasmid pET11c vector (Novagen) [3,31]. The 163-residue catalytic domain of MMP-12 (MMP-12_CAT) was expressed in *E. coli* BL21 (DE3) cells transformed with the expression plasmid. Cells were cultured at 37°C in LB medium supplemented with 100 ug/ml of ampicillin. Protein expression was induced when the cells had grown to an OD 600 value of 0.8 by the addition of IPTG to a final concentration of 1 mM. Cells were harvested 4 hours post-induction by centrifugation at 5,000 g at 4°C for 20 minutes, and the pellets were stored at −20°C.

### Isolation of pure EGFP and MMP-12_CAT inclusion bodies from *E. coli* cells

Cell pellets were thawed briefly at room temperature and resuspended in 30 ml of PBS buffer. The mixture was sonicated on ice until a homogeneous suspension was formed. The resulting cell lysate was centrifuged at 12,000 g for 20 minutes. The supernatant (S) was retained for analysis by SDS–PAGE, the insoluble inclusion bodies (P) were resuspended in 30 ml of washing buffer (20 mM Tris, 300 mM NaCl, 1 mM EDTA, 1% Triton X-100, 1 M urea, pH 8.0) and also analyzed by SDS–PAGE. In order to obtain pure EGFP and MMP-12_CAT inclusion bodies, the pellets were washed extensively for three times with 30 ml of washing buffer. Finally, the inclusion bodies were washed with PBS buffer to remove contaminating detergent and centrifuged at 12,000 g for 20 minutes and the purified inclusion bodies were used for subsequent solubilization and refolding.

### Comparative solubilization of EGFP and MMP-12_CAT inclusion bodies by two different methods

In order to compare the solubilization power of freeze-thawing method with that of urea-denatured method, the same amount of purified EGFP and MMP-12_CAT inclusion body pellets was resuspended in PBS buffer at pH 8 containing different molar concentration of urea (0-8 M). For the traditional urea-denatured method, the suspension was stirred for 30 minutes at room temperature and centrifuged (12,000 g, 4°C, 15 minutes) to collect the supernatant. For the freeze-thawing method, the suspension was frozen at −20°C and thawed at room temperature, centrifuged at 12,000 g for 15 minutes at 4°C to collect the supernatant. Supernatants (10ul) were analyzed by SDS–PAGE to check the quality of the protein. Protein concentration was estimated by Micro BCA Protein Assay Kit (Thermo) and BSA was used as standard for the estimation of protein concentration (Table 1).

### Solubility of EGFP and MMP-12_CAT inclusion bodies in different solvents

Five different solvents in presence of 2 M urea were employed to solubilize EGFP and MMP-12_CAT inclusion bodies: PBS (pH 8), 20 mM sodium phosphate (NaP, pH 8), 20 mM potassium phosphate (KP, pH 8), 20 mM Tris–HCl (pH 8), and deionized water (pH 8.5). Homogenous inclusion body suspension in PBS buffer was centrifuged and the same volume of each of the above solubilizing solvents was added to the pellets. Suspension was frozen at −20°C for overnight and thawed at room temperature, and was centrifuged at 12,000 g for 15 minutes at 4°C to get clear supernatant. Supernatants (5ul) were analyzed by SDS–PAGE to check the quality of the protein. Protein bands were quantitated by densitometric analysis using ImageJ Software (National Institutes of Health, Bethesda, MD).

### Solubility of EGFP and MMP-12_CAT inclusion bodies in potassium phosphate buffer at different pH

Purified EGFP and MMP-12_CAT inclusion bodies were solubilized in 20 mM potassium phosphate buffer at different pHs (5–10) in the presence of 2 M urea. Homogenous inclusion body suspension in potassium phosphate buffer at different pHs was frozen at −20°C and thawed at room temperature, centrifuged at 12,000 g for 15 minutes at 4°C to get clear supernatant. Supernatants (5ul) were analyzed by SDS–PAGE. Protein bands were quantitated by densitometric analysis using ImageJ Software.

### Optimization of freeze temperature for solubilization of EGFP and MMP-12_CAT inclusion bodies

Homogenous suspension of EGFP and MMP-12_CAT inclusion bodies was solubilized in 20 mM potassium phosphate buffer at pH 8 containing 2 M urea. The suspension was frozen at −20°C, −40°C and −80°C for overnight, respectively. Then, the suspension was thawed at room temperature and centrifuged (12,000 g, 4°C, 15 minutes) to collect the supernatant. Supernatants (5ul) were also analyzed by SDS–PAGE. Protein bands were quantitated by densitometric analysis using ImageJ Software.

### Recovery of bioactive EGFP and MMP-12_CAT from inclusion bodies

The solubilized EGFP from inclusion bodies in PBS buffer at pH 8 containing 2 M urea from freeze-thawing method and 8 M urea from traditional urea-denatured method were refolded by rapid dilution. This was done by adding solubilized proteins drop wise into PBS buffer at pH 8 until a final urea concentration of 1 M and stored at 4°C for one week.

Fractions containing solubilized MMP-12_CAT from inclusion bodies in PBS buffer at pH 8 containing 2 M urea (from freeze-thawing method) and 8 M urea (from traditional urea-denatured method) were used to obtain active forms. To avoid precipitation shown up, the concentration of MMP-12_CAT was adjusted to 0.5 mg/ml, and then placed into a MWCO 5 kDa dialysis membrane, and then diluted into refolding buffer (20 mM Tris, 100 mM NaCl, 10 mM CaCl2, 0.2 Mm ZnCl2, pH 7.5). Each dialysis step was performed at 4°C for 12 hours against ten volumes of the dialysis buffer and three buffer changes in each step.

### Fluorescence and Circular Dichroism spectra of EGFP samples

Fluorescence emission spectra of EGFP were recorded using a Synergy H4 Hybrid Microplate Reader (Biotek). The fluorescence spectra of five EGFP samples at 0.5 mg/ml concentrations were measured, including soluble expressed EGFP, two unfolded EGFP and two refolded EGFP. EGFP samples were excited at 450 nm and emission spectra were collected from 480–570 nm.

To investigate the secondary structure contents of EGFP samples obtained from freeze-thawing method, CD spectra of solublilized EGFP (5 μM of protein concentration in 20 mM Tris–HCl with 2 M urea) were recorded using J-815 spectro-polarimeter (Jasco) in the wavelength range of 190–250 nm at 25°C. The photomultiplier voltage read never exceeded 600 V in the spectral regions. Each spectrum was scanned five times and the average spectrum was plotted. The cuvette path length was 1 mm for far-UV region measurements with a step size of 0.5 nm and a bandwidth of 1 nm.

### Fluorescent enzymatic assay of refolded MMP-12_CAT

The activity of two refolded MMP-12_CAT samples was determined using the fluorometric substrate Mca-PLGLEEA-Dpa-NH2 (ANASPEC) (27). Reactions (200 ul) were performed at 25°C, using a substrate concentration of 8 um and 5 nm MMP-12_CAT in assay buffer (50 mM Tris–HCl, 10 mM CaCl_2_, 0.05% Brij-35 (v/v), pH 7.4) in black 96-well plates (Corning). The release of the fluorescent product McaPL was monitored continuously using a Synergy H4 Hybrid Microplate Reader (Biotek). The excitation filter used was 325 nm with a bandwidth of 20 nm and the emission filter was 393 nm with a bandwidth of 20 nm. The selected gain was 4 with readings every 1 minute for 14 cycles, where the cleavage of substrate was linear with respect to time.

### Expression of five more inclusion body proteins

The plasmids containing the genes of five proteins were transferred into the BL21 (DE3) strain. The cells were grown in LB medium at 37°C. All over-expressions were induced by 1 mM IPTG at 37°C for 4 hours. The cells were harvested at 5,000 g at 4°C for 15 minutes, lysed with 30 ml of PBS buffer by sonication on ice, and centrifuged (12,000 g, 4°C, 20 minutes). The pellets were cleaned three times with 30 ml of washing buffer and centrifuged (12,000 g, 4°C, 15 minutes). The purified five inclusion body proteins were suspended in PBS buffer containing 2 M urea at pH 8. Before freeze-thawing, 5ul of suspension (T) was retained for analysis by SDS-PAGE, then, the suspension was frozen at −20°C for overnight and thawed at room temperature, centrifuged at 12,000 g for 15 minutes at 4°C to collect the supernatant. The supernatants (5ul) were also analyzed by SDS–PAGE to estimate the percent solubilization of inclusion bodies.

## Abbreviations

EGFP: enhanced green fluorescent protein
MMP-12_CAT: the catalytic domain (residues Gly106 to Asn268) of human macrophage metalloelastase (MMP-12)
